# Establishment of BmoR-based biosensor to screen isobutanol overproducer

**DOI:** 10.1186/s12934-019-1084-2

**Published:** 2019-02-07

**Authors:** Huan Yu, Ning Wang, Wenbo Huo, Yuhong Zhang, Wei Zhang, Yu Yang, Zhenya Chen, Yi-Xin Huo

**Affiliations:** 1 Key Laboratory of Molecular Medicine and Biotherapy, School of Life Science, Beijing Institute of Technology, No. 5 South Zhongguancun Street, Beijing, 100081 China; 2 Biotechnology Research Institute of Chinese Academy of Agricultural Sciences, No. 12 South Zhongguancun Street, Beijing, 100081 China

**Keywords:** BmoR, Biosensor, Screening, Isobutanol, Mutagenesis

## Abstract

**Background:**

Isobutanol, a C4 branched-chain higher alcohol, is regarded as an attractive next-generation transport fuel. Metabolic engineering for efficient isobutanol production has been achieved in many studies. BmoR, an alcohol-regulated transcription factor, mediates a σ^54^-dependent promoter *P*_*bmo*_ of alkane monooxygenase in *n*-alkane metabolism of *Thauera butanivorans* and displays high sensitivity to C4–C6 linear alcohols and C3–C5 branched-chain alcohols. In this study, to achieve the high-level production of isobutanol, we established a screening system which relied on the combination of BmoR-based biosensor and isobutanol biosynthetic pathway and then employed it to screen isobutanol overproduction strains from an ARTP mutagenesis library.

**Results:**

Firstly, we constructed and verified a GFP-based BmoR-*P*_*bmo*_ device responding to the isobutanol produced by the host. Then, this screening system was employed to select three mutants which exhibited higher GFP/OD_600_ values than that of wild type. Significantly, GFP/OD_600_ of mutant 10 was 190.7 ± 4.8, a 1.4-fold higher value than that of wild type. Correspondingly, the isobutanol titer of that strain was 1597.6 ± 129.6 mg/L, 2.0-fold higher than the wild type. With the overexpression of upstream pathway genes, the isobutanol production from mutant 10 reached 14.0 ± 1.0 g/L after medium optimization in shake flask. The isobutanol titer reached 56.5 ± 1.8 g/L in a fed-batch production experiment.

**Conclusions:**

This work screened out isobutanol overproduction strains from a mutagenesis library by using a screening system which depended on the combination of BmoR-based biosensor and isobutanol biosynthetic pathway. Optimizing fermentation condition and reinforcing upstream pathway could realize the increase of isobutanol production from the overproducer. Lastly, fed-batch fermentation of the mutant enhanced the isobutanol production to 56.5 ± 1.8 g/L.

## Background

Higher alcohols including *n*-butanol, isobutanol, 2-methyl-1-butanol (2-MB), 3-methyl-1-butanol (3-MB) and 2-phenylethanol have drew much attention as next-generation transport fuels because of their higher energy density, lower vapor pressure and lower hygroscopicity when compared with traditional biofuel such as ethanol [1]. Microbial-based metabolic engineering, as an eco-friendly strategy, has been used to produce many value-added compounds from renewable resources [2–7]. Microbial production of higher alcohols also has been realized in many engineered hosts [3, 8–10]. The pathways for the biosynthesis of these alcohols were extended from 2-keto acids, the intermediates in amino acid biosynthetic pathways. Subsequently, 2-keto acids were converted to corresponding alcohols by the sequential catalysis through 2-keto acid decarboxylase (KDC) and alcohol dehydrogenase (ADH) [11, 12].

**Fig. 1:**
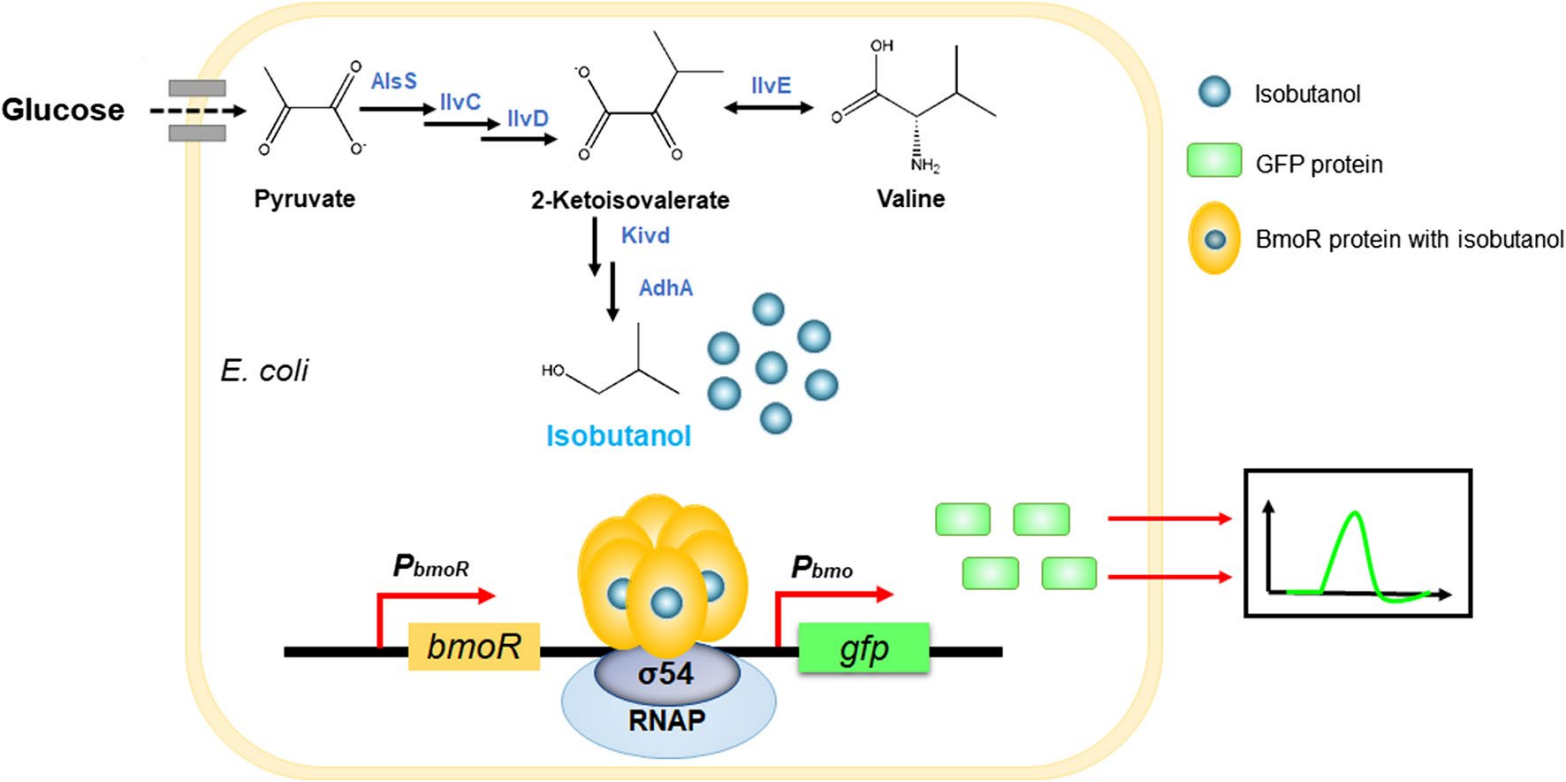
The pathway for isobutanol production and mechanism of BmoR-based biosensor response to isobutanol via detection of GFP fluorescence. AlsS, acetolactate synthase; IlvC, acetohydroxy acid isomeroreductase; IlvD, dihydroxy-acid dehydratase; IlvE, branched-chain-amino-acid transaminase; Kivd, ketoisovalerate decarboxylase; AdhA, alcohol dehydrogenase. *P*_*bmoR*_, a constitutive promoter to regulate the BmoR expression. Promoter *P*_*bmo*_ drove the GFP expression and was regulated by BmoR protein. Isobutanol generated from glucose bound to BmoR and then activated Eσ^54^ closed complex to induce the transcription initiation of *P*_*bmo*_

The precursor for isobutanol biosynthesis was 2-ketoisovalerate (2-KIV), an intermediate in l-valine biosynthetic pathway which could be biosynthesized from pyruvate through a three-step enzymatic catalysis by the enzymes AlsS, IlvC and IlvD (Fig. 1). The conversion between 2-KIV and l-valine was catalyzed by an endogenous aminotransferase. So far, many microorganisms such as *Escherichia coli* (*E. coli*), *Bacillus subtilis* (*B. subtilis*), *Saccharomyces cerevisiae* (*S. cerevisiae*) and *Corynebacterium glutamicum* (*C. glutamicum*) have been engineered to produce isobutanol [8, 13–15]. In these studies, the ketoisovalerate decarboxylase (Kivd) and alcohol dehydrogenase (AdhA) would catalyze 2-KIV to isobutyraldehyde and convert isobutyraldehyde to isobutanol, respectively. Hence, assembling the endogenous 2-KIV biosynthetic pathway and exogenous Kivd and AdhA could realize the biosynthesis of isobutanol from glucose. To broaden the carbon source for higher alcohols production, Huo et al. employed single-cell protein wastes as carbon source and reached 56% of the theoretical yield [16].

In general, modifying microorganism was an essential step in metabolic engineering. Microbial mutation breeding was a common strategy which included random mutagenesis by nitrosoguanidine (NTG) [17], ultraviolet treatment (UV) [18] or atmospheric and room temperature plasma (ARTP) [19, 20]. After mutagenesis of the host microorganism, the methods for screening mutant strains that overproduced the target molecules or their relevant precursors were desired. Traditional screening methods depended on high performance liquid chromatography (HPLC) or gas chromatograph (GC), which were time-consuming and limited by high cost and low efficiency. Biosensor, as an emerging tool, could respond to the specific metabolite and then dynamically regulate the whole metabolic pathway, leading to the balance of metabolic flux and the increase of the productivity of desired compound [21–26]. Biosensors were used in many studies to regulate the expressions of fluorescent proteins, which provided the detection signals for high-throughput screening [27–29]. Zhang et al. constructed NCgl0581 biosensor in *C. glutamicum* to screen out a high-producing l-serine strain that produced 34.78 g/L l-serine with a yield of 0.35 g/g sucrose, which were 35.9 and 66.7% higher than those of the parent strain [30]. Liu et al. adopted the l-phenylalanine-specific transcription factor (TyrR) in *E. coli* to screen out a variant which could yield 1.8-fold higher l-phenylalanine when compared with the parent strain [31].

So far, using a strategy for screening high-level isobutanol production strains from the mutagenesis library has not been accomplished. BmoR, an alcohol-regulated transcription factor in *n*-alkane metabolism of *Thauera butanivorans*, mediated a σ^54^-dependent promoter *P*_*bmo*_ of alkane monooxygenase [32]. The alcohol molecules could bind to BmoR (an enhancer-binding protein as hexamer) and the generated combination would remodel σ^54^-RNAP holoenzyme (Eσ^54^) closed complex and then activate the transcription initiation of promoter *P*_*bmo*_ [33]. Dietrich et al. firstly used BmoR as a biosensor to respond to various alcohols and the results showed that BmoR exhibited high sensitivity to C4–C6 linear alcohols and C3–C5 branched-chain alcohols, of which *n*-butanol showed the broadest linear range of detection (from 100 μM to 40 mM). Then, BmoR-based biosensor was employed to screen out the strain with the highest conversion rate from 2-oxopentanoate to *n*-butanol in a RBS mutagenesis library [34].

For the purpose of acquiring the high-level isobutanol production strains, it is essential to acquire efficient host microorganisms. In this study, a biosensor-based strategy was designed for screening isobutanol overproduction strains. Isobutanol generated from glucose in hosts or absorbed from environment would bind to BmoR hexamer whose expression was driven by a constitutive promoter. The BmoR with isobutanol binding would activate Eσ^54^ closed complex and then induce the transcription initiation of promoter *P*_*bmo*_. The whole response process was shown in Fig. 1. Firstly, we constructed a GFP-based BmoR-*P*_*bmo*_ biosensor system and testified its effectiveness. Then, this biosensor system combining with isobutanol biosynthetic pathway was introduced into the mutagenesis library to screen the isobutanol overproducers. As a result, the mutant which was screened out from the mutagenesis library could produce 14.0 ± 1.0 g/L isobutanol in shake flask and 56.5 ± 1.8 g/L isobutanol in a 3-L bioreactor. This work illustrated that BmoR-based biosensor could respond to intracellular isobutanol and could be used to screen isobutanol overproducers, which has the potential to be engineered to overproduce l-valine since the biosynthesis pathways of isobutanol and l-valine shared the same precursor 2-KIV.

## Results and discussion

### Characterization of BmoR-*P*_*bmo*_ biosensor via feeding isobutanol

**Fig. 2:**
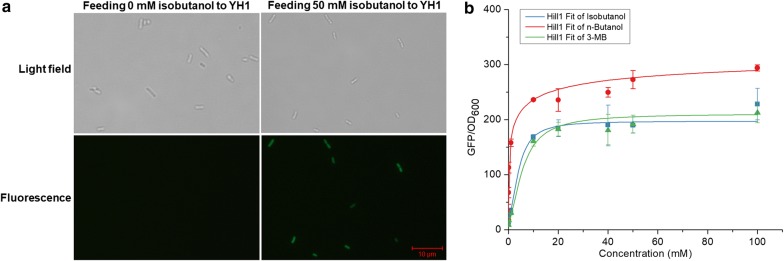
Fluorescence distribution of the cells and the response of BmoR-based biosensor to different alcohols which were fed into the culture. Strain YH1 (BW25113 (F′) harboring plasmid pYH1) was used for fluorescence detection. For **a**, the fluorescence microscope (Nikon model Eclipse Ni-U) was used to observe fluorescence distribution of the cells. Fluorescence was not observed when feeding 0 mM isobutanol to the culture and was observed when feeding 50 mM isobutanol. For **b**, the response values of this biosensor towards isobutanol, *n*-butanol and 3-MB which were represented by blue, red and green lines, respectively

To verify the response of the BmoR-based biosensor to alcohols, *gfp* was chosen as a reporter gene under the control of promoter *P*_*bmo*_. *E. coli* BW25113 (F′) harboring plasmid pYH1 (YH1) was used for subsequent experiments. To conduct the experiments, 50 μL pre-culture of strain YH1 and 950 μL fresh LB medium supplemented with different final concentrations (0, 0.01, 0.1, 1, 10, 20, 40, 50 or 100 mM) of *n*-butanol, isobutanol and 3-MB, respectively, were added into in 96-deep-well plates and the cultures were then left at 30 °C incubator for 16 h. Then, microplate reader was used to detect the GFP fluorescence and OD_600_ values. The fluorescence microscope (Nikon model Eclipse Ni-U) was used to observe fluorescence distribution of the cells. As the results shown in Fig. 2a, the fluorescence distributed evenly in the respective cell and all the cells had the similar fluorescence intensity when feeding 50 mM isobutanol to the culture. The response which represented as GFP/OD_600_ values were shown in Fig. 2b. The response of this biosensor was improved along with the increase of the concentration of alcohols. The response of this biosensor to *n*-butanol was higher than the response of other two alcohols whatever the concentration of the alcohols was. The response of the biosensor in the presence of isobutanol or 3-MB feeding was weak if the concentations of the alcohols were lower than 1 mM. When the concentration of isobutanol was increased to 10 mM, the GFP/OD_600_ value was 168.6 ± 4.13, a similar value to 3-MB but was 1.4-fold lower when compared with *n*-butanol. The GFP/OD_600_ towards isobutanol reached 228.2 ± 28.5 when the isobutanol concentration reached 100 mM. Further enhancing the isobutanol concentration would inhibit the cell growth and then resulted in the decrease of response, similar as the other two alcohols. Table 1 displayed the *K*_m_ of this biosensor towards these three alcohols. This biosensor has a *K*_m_ of 4.2 ± 0.3 mM towards isobutanol which was close to 3-MB and 1.8-fold higher than *n*-butanol. Besides, blue–white screening experiment was conducted to confirm the response of BmoR-based biosensor towards alcohols which was fed to the culture. The single colonies of *E. coli* BW25113 (F′) harboring pYH2 (YH2) which spread on the LB plate with different concontrations of isobutanol and 100 μg/mL X-gal adding were not able to turn blue (data not shown), probably due to the evaporation of isobutanol. Taken together, this biosensor could respond to *n*-butanol, isobutanol and 3-MB and had significant response when the concentration of alcohols exceeded 1 mM.

**Table 1:** GFP/OD_600max_ and *K*_m_ values of BmoR-based biosensor towards isobutanol, *n*-butanol and 3-MB

Compound	Dynamic range (GFP/OD_600max_)	*K*_m_ (mM)
Isobutanol	197.8 ± 51.2	4.2 ± 0.3
*n*-Butanol	336.8 ± 137	2.3 ± 1.1
3-MB	200.7 ± 7.5	4.6 ± 1.1

### Optimizing the copy number of BmoR-*P*_*bmo*_ device in the isobutanol-producing strains

**Fig. 3:**
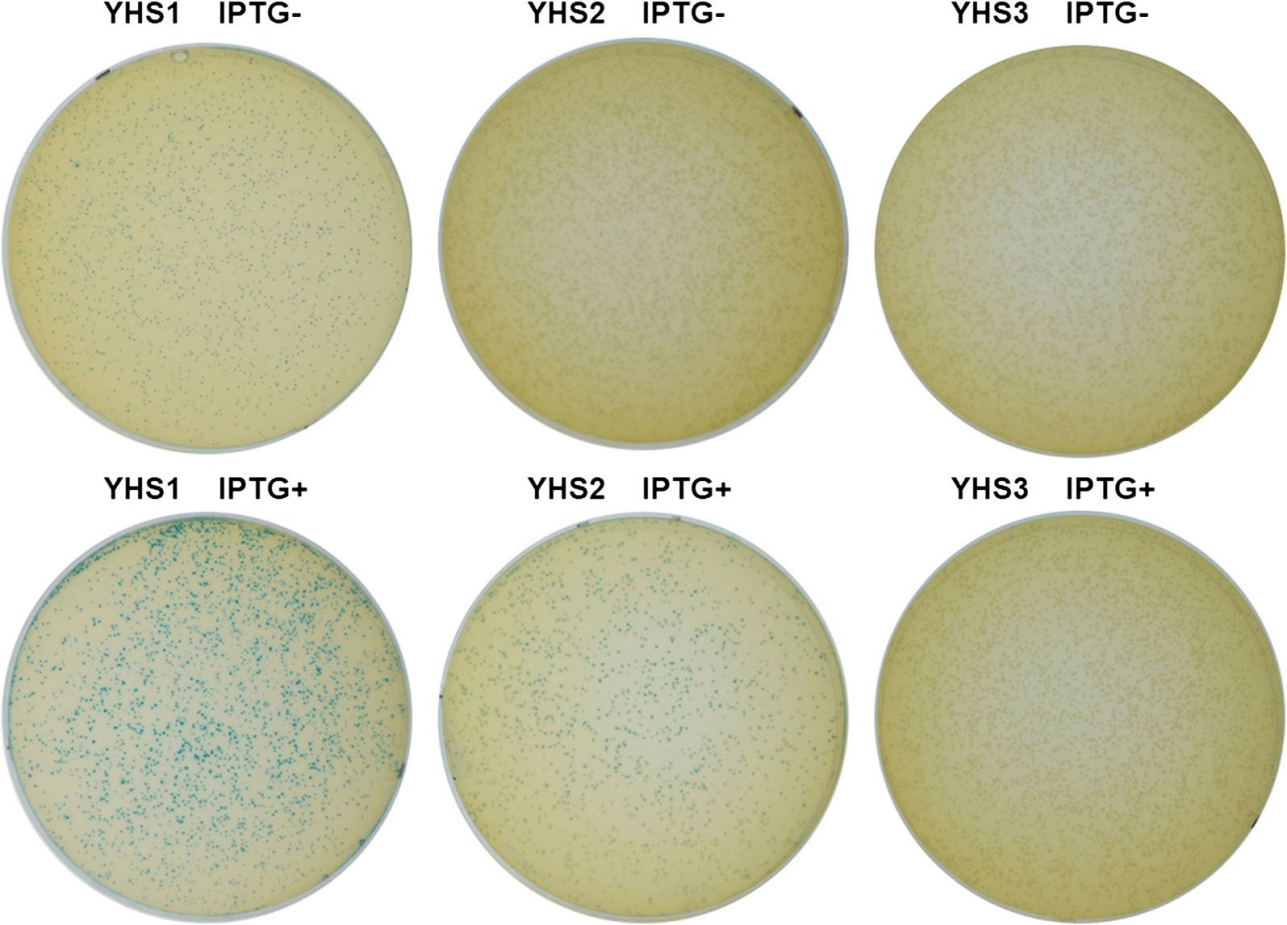
Optimization of copy number of the plasmid for BmoR-*P*_*bmo*_ device construction via blue–white screening experiments. The BmoR-*P*_*bmo*_ device in the strain YHS1 (BW25113 (F′) harboring pSA69 and pYH4) was constructed in a high-copy-number plasmid. The BmoR-*P*_*bmo*_ device in the strain YHS2 (BW25113 (F′) harboring pSA69, pSA65 and pYH5) was constructed in a medium-copy-number plasmid. The BmoR-*P*_*bmo*_ device in the strain YHS3 (BW25113 (F′) harboring pSA69, pSA65 and pYH3) was constructed in a low-copy-number plasmid

The blue–white screening experiment was performed on the strains YHS1, YHS2 and YHS3 which have the BmoR-based biosensor and the ability of producing isobutanol. As shown in Fig. 3, YHS1 containing BmoR-*P*_*bmo*_ device in a high-copy-number plasmid turned blue on the X-gal plates without IPTG induction, illustrating that high-copy-number plasmid were not suitable for this biosensor construction due to the leaking expression of *P*_*L*_*lacO*_*1*_ promoter. On the contrary, the colonies of strain YHS3 which containing BmoR-*P*_*bmo*_ device in a low-copy-number plasmid were unable to turn blue in spite of IPTG inducing. Fortunately, the colonies of strain YHS2 containing BmoR-*P*_*bmo*_ device in a medium-copy-number plasmid could only turn blue in the presence of IPTG induction, suggesting the medium-copy-number plasmid was optimal for constructing BmoR-*P*_*bmo*_ device. Subsequently, the BmoR-*P*_*bmo*_ device in medium-copy-number plasmid was used for the following experiments.

### Establishment of the screening system

**Fig. 4:**
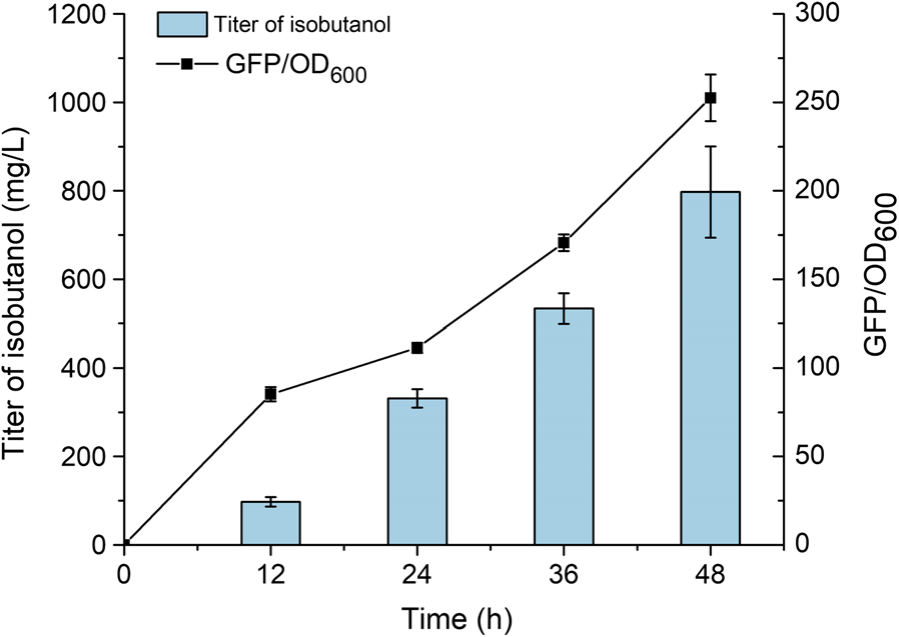
Verification of the relevance between isobutanol production and GFP/OD_600_ value of the host strain. For this experiment, strain YHS5 (BW25113 (F′) harboring pSA65 and pYH10) which have the BmoR-based biosensor and the ability to produce isobutanol was used

For conducting the subsequent screening experiment, we firstly established a screening system based on blue–white screening. Blue–white screening experiment was carried out in strain YHS4 which did not contain pSA69, an enhancing precursor production plasmid and the results showed the colonies were able to turn blue though the strain was wild-type (data not shown). Based on this result, we concluded the blue–white screening was not suitable for screening the high-level isobutanol production strains from the ARTP mutant library. In this study, we also constructed a GFP fluorescence-based screening system. Plasmid pSA65 containing *kivd* and *adhA* genes which were under the control of an IPTG-inducible promoter *P*_*L*_*lacO*_*1*_ and used for conversion of 2-ketoisovalerate to isobutanol and plasmid pYH10 containing BmoR-*P*_*bmo*_ device were introduced into *E. coli* BW25113 (F′) together to obtain strain YHS5. As the results shown in Fig. 4, the GFP/OD_600_ value was increased consistently throughout the fermentation process (48 h), which was in sync with the raise of isobutanol titer. The strain reached a highest GFP/OD_600_ value (252.6 ± 13.2) at 48 h, while 797.3 ± 103.1 mg/L isobutanol was accumulated during the same period. These results suggested that this GFP fluorescence-based system could be applied for the following screening experiments.

### Screening high-level isobutanol production strains

**Fig. 5:**
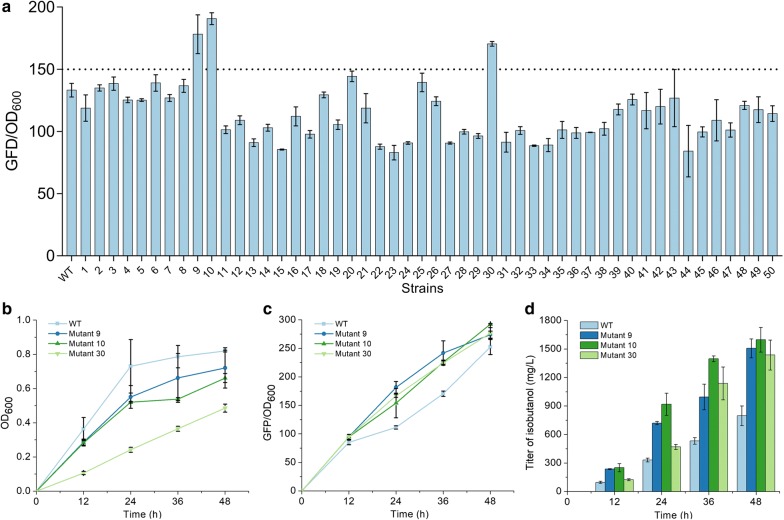
Screening the high-level isobutanol-producing strain via BmoR-based biosensor. For **a**, screening strains from ARTP mutagenesis library. For **b**, the growth curves of mutants (mutant 9, 10 and 30) and wild type. For **c**, the GFP/OD_600_ values of mutants and wild type. For **d**, isobutanol productions of the mutants and wild type

A mutant library of *E. coli* BW25113 (F′) was obtained by using the ARTP method. The mutated cells were co-transformed with plasmids pYH10 and isobutanol-producing plasmid pSA65, and 200 single colonies of these engineered strains were inoculated into M9 medium individually after pre-incubation in LB. The M9 medium was without yeast extract because the l-valine in yeast extract could be as precursor for isobutanol production which might interfere the screening process. After measuring GFP/OD_600_ value of every colony, we screened out three strains, mutant 9, 10 and 30, which exhibited GFP/OD_600_ values of 178.2 ± 15.6, 190.7 ± 4.8 and 170.5 ± 1.9, respectively (Fig. 5a). The GFP/OD_600_ values of mutant 9, 10 and 30 were 1.3, 1.4 and 1.3-fold higher GFP/OD_600_ values than that of wild type (YHS5), respectively. According to these results, we speculated that these three mutants might be able to produce more isobutanol than wild type in the absence of overexpression of upstream pathway for precursor 2-KIV production. Subsequently, shake flask experiment was adopted to test the isobutanol production of these three mutated strains. The growth curves of the mutants were shown in Fig. 5b. The trend of the growth curves of mutant 9, 10 and 30 were similar to that of wild type and the cell growths were increased continuously within 48 h. Significantly, the OD_600_ values of mutant 9, 10 and 30 were lower than that of wild type during the whole fermentation process. Mutant 30 had an OD_600_ value of 0.5 ± 0.02 at 48 h, a 1.7-fold lower value than that of wild type at the same time point. The OD_600_ value of mutant 10 at 48 h was 0.7 ± 0.1 which was close to that of wild type. These results suggested that the mutagenesis affected the growth of *E. coli* BW25113 (F′). Fig 5c, d showed that the GFP/OD_600_ values and the isobutanol productions of wild type and mutants during the fermentation, respectively. The GFP/OD_600_ values were increased with the isobutanol titer. The producing of isobutanol in these strains was continuous during the whole fermentation process and the isobutanol titer attained the highest value at 48 h. As expected, all the mutants could produce more isobutanol than wild type at any point in the fermentation process, which corresponding to their GFP/OD_600_ value. Within 48 h, 1506.7 ± 99.3, 1597.6 ± 129.6 and 1436.8 ± 157.7 mg/L isobutanol were accumulated in the cultures of mutant 9, 10 and 30, respectively. Remarkably, the isobutanol titer of mutant 10 was highest among these three mutants, representing a 2.0-fold higher value when compared with that of wild type. These results demonstrated that BmoR-*P*_*bmo*_ device could be used as an efficient tool to screen high-level isobutanol-producing strains via measuring GFP fluorescence.

### Improvement of isobutanol production via strengthening the upstream pathway and optimizing yeast extract concentration

**Fig. 6:**
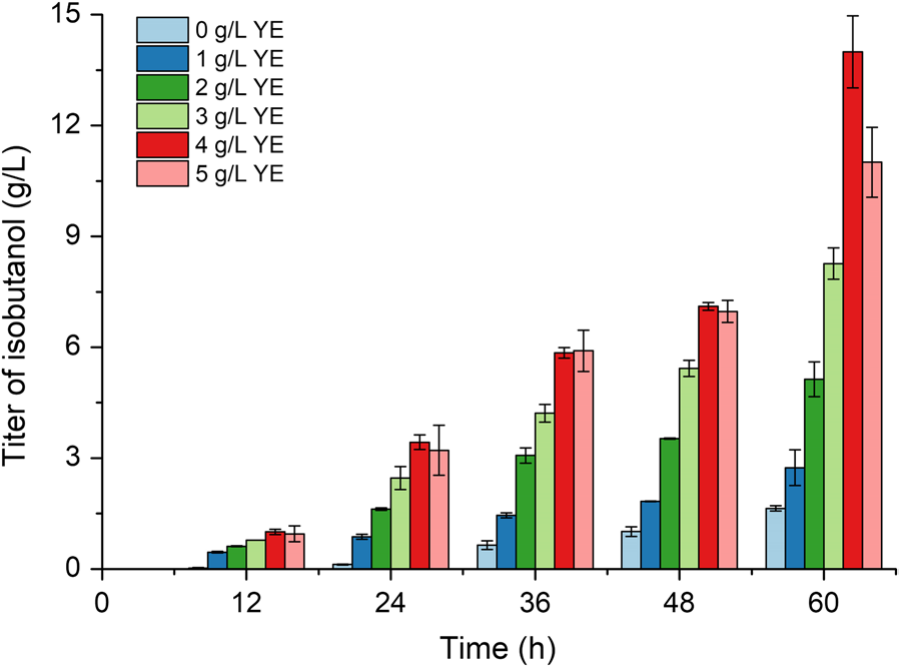
Optimization of yeast extract concentration for isobutanol production. For this experiment, strain YHS6 (mutant 10 harboring plasmid pSA69) was used

Based on the above results, mutant 10 was selected as a candidate to achieve the overproduction of isobutanol in shake flask. Firstly, to enhance the supply of precursor (2-KIV), plasmid pSA69 was introduced into mutant 10 (strain YHS6) to express AlsS, IlvC and IlvD which included in the 2-ketoisovalerate biosynthetic pathway. High concentration of yeast extract was beneficial for heterologous protein expressions, which might improve the desired chemical production. On the other hand, the fast cell growth facilitated by the high amount of yeast extract would consume more carbon sources, leading to the decrease of desired compound production. Therefore, optimization of yeast extract concentration was an essential step for enhancing the isobutanol production. M9 medium with 40 g/L glucose and different concentrations of yeast extract (0, 1, 2, 3, 4 or 5 g/L) was used for the fermentation of strain YHS6. As the results shown in Fig. 6, the isobutanol titer increased along the time course regardless of the yeast extract concentration. Besides, the production of isobutanol was improved continuously with the raise of yeast extract concentration until the yeast extract concentration reached 4 g/L. With 4 g/L yeast extract adding into the medium, the isobutanol titer attained 14.0 ± 1.0 g/L at 60 h, which was 8.8-fold higher than that of mutant 10 without optimization. Further increasing yeast extract concentration to 5 g/L did not enhance the isobutanol production. These results suggested that reinforcing the upstream pathway and optimizing yeast extract concentration was effective to improve isobutanol biosynthesis.

### Production of isobutanol in a fed-batch bioreactor

**Fig. 7:**
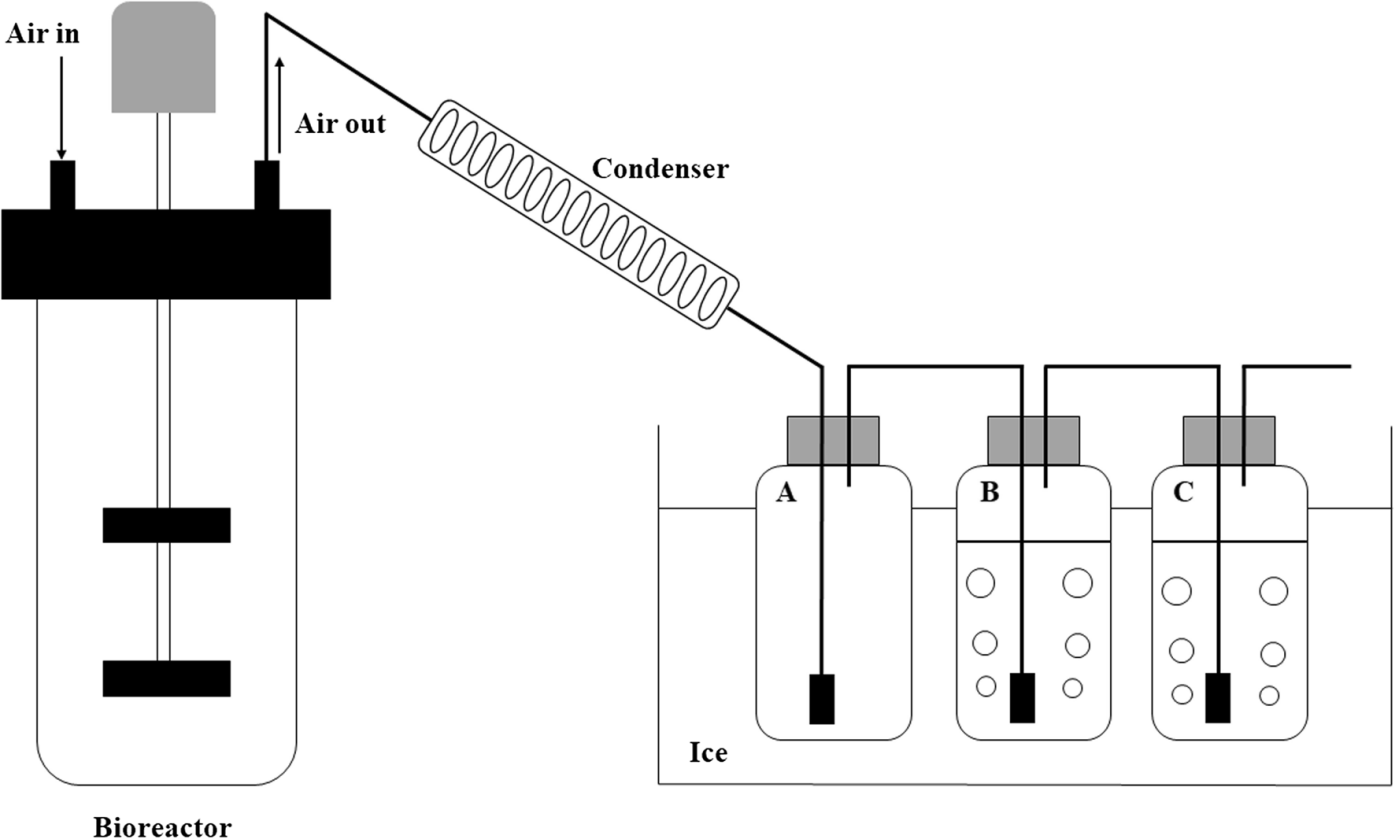
Schematic diagram of the fed-batch fermentation equipment for isobutanol production by strain YHS6 (mutant 10 harboring plasmid pSA69). Bottle A was empty and cooled with ice to collect the condensed isobutanol. Bottles B and C containing 800 mL water was also cooled with ice to collect the residual uncondensed isobutanol

**Fig. 8:**
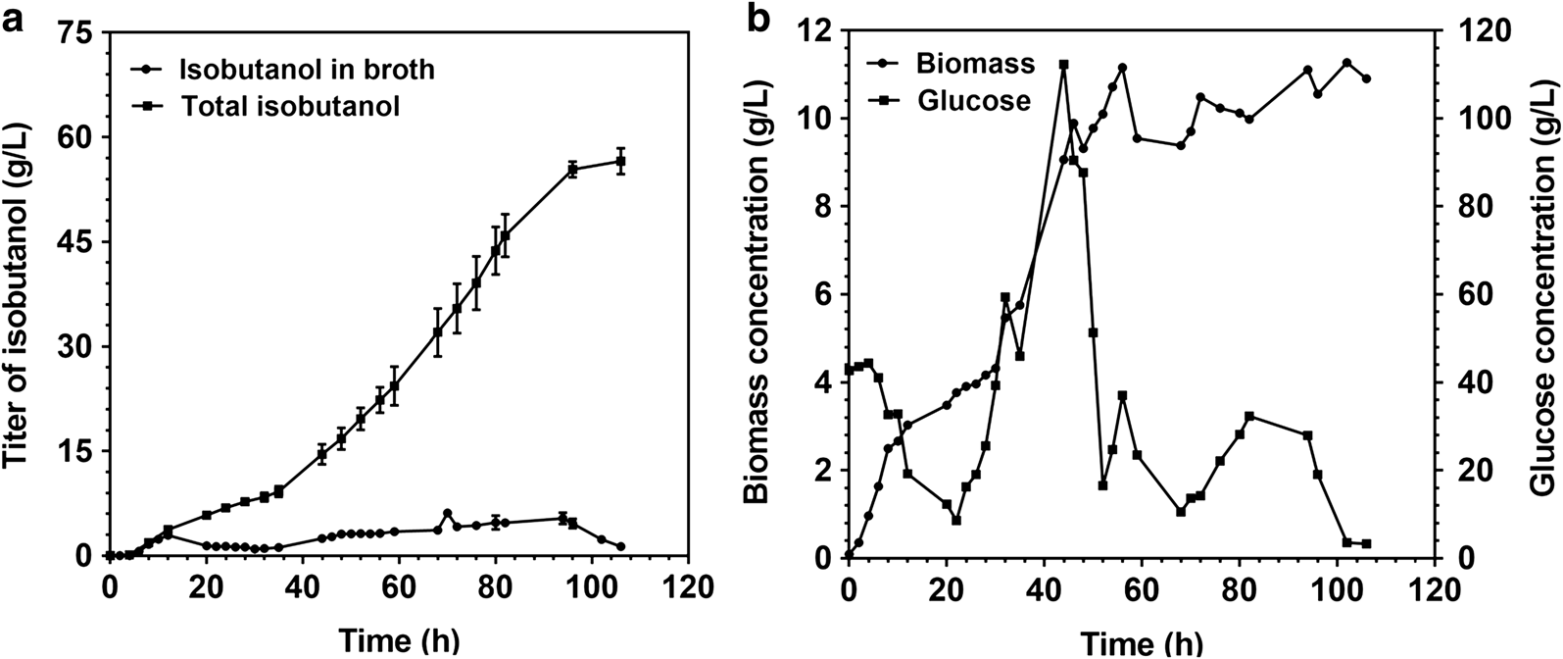
Typical kinetics of isobutanol production by strain YHS6 (mutant 10 harboring plasmid pSA69) in a 3-L bioreactor at 30 °C. For **a**, total isobutanol production which was calculated as sum of isobutanol in collection bottles A, B, C and the broth with a working volume of 2 L, and the isobutanol production in broth. For **b**, cell growth and glucose consumption during the whole fermentation process

A 3-L bioreactor was employed to examine the potential of the isobutanol production capability of mutant 10. To conduct this experiment, the strain YHS6 (mutant 10 harboring plasmid pSA69) was used. The schematic diagram of the fed-batch fermentation equipment was shown in Fig. 7. The isobutanol which yielded by the YHS6 in the broth would be stripped out and condensed by the condenser, and then was collected into the bottle A, B and C. The results of fed-batch culture were shown in Fig. 8. Figure 8a displayed the total isobutanol production which was calculated as the sum of isobutanol in collection bottle A, B, C and the broth with a working volume of 2 L, and the isobutanol production in the broth. In the first 12 h after induction, the isobutanol was produced continuously and the titer reached 2.9 ± 0.3 g/L in the broth at 12 h. At the same time point, the total of isobutanol production was 3.7 ± 0.3 g/L, suggesting that the large proportion of isobutanol was remained in the broth which might inhibit the growth of the cells. Based on that, the air flow rate was raised to 3 vvm (the abbreviation of air volume/culture volume/min) in order to remove isobutanol from the broth. In 20–52 h, the isobutanol titer in the broth was maintained less than 3 g/L. In the subsequent fermentation (56–82 h), the isobutanol titer in the broth was increased and reached 4.7 ± 0.6 g/L at 82 h. At the end of the fermentation (106 h), the isobutanol titer in the broth was only 1.3 ± 0.1 g/L and the total isobutanol titer reached 56.5 ± 1.8 g/L, which was 4.0-fold higher than that of shake flask experiment. In the previous reported study, the wild-type strain *E. coli* BW25113 (F′) containing isobutanol production pathway could accumulate 41.4 ± 4.5 g/L isobutanol in 72 h in a 1-L bioreactor [35]. Compared with the above reported study, the isobutanol titer in this present study was 1.4-fold higher than that of *E. coli* BW25113 (F′). This result illustrated that we used the BmoR-based biosensor to screen out a promising strain which could be applied for high-level isobutanol production. Fig 8b showed the biomass and glucose concentration at different time point in the whole fermentation process. The strain YHS6 reached the maximum cell density at 56 h. After this time point, the cells stopped growing and the cell density was maintained at a relatively constant value. During the fermentation process, the stock solution containing 500 g/L glucose was fed to the culture to maintain the glucose concentration in broth above 5 g/L because the glucose was the main substrate for isobutanol production [8] and the low glucose concentration for a long time would result in the decrease of the isobutanol production.

## Conclusions

In this work, we confirmed that the biosensor BmoR could respond to isobutanol and could be utilized as a basic element for screening isobutanol-overproducing strains. We assembled a BmoR-*P*_*bmo*_ system and then combined it with isobutanol biosynthetic pathway to construct a GFP-based screening strategy in *E. coli*. From an ARTP mutagenesis library, we screened out three strains which have ability to produce more isobutanol than wild type in the absence of overexpression of upstream pathway for precursor 2-KIV production. Besides, reinforcing the 2-KIV overproduction pathway and optimizing medium improved the isobutanol titer from a mutated host to 14.0 ± 1.0 g/L. Moreover, the titer of isobutanol reached 56.5 ± 1.8 g/L in a fed-batch production experiment.

## Methods

### Medium, strains and plasmids

LB (10 g/L tryptone, 5 g/L yeast extract and 10 g/L NaCl) medium was used for cell incubation and plasmid construction. M9 medium which contains 6 g/L Na_2_HPO_4_, 3 g/L KH_2_PO_4_, 1 g/L NH_4_Cl and 0.5 g/L NaCl, 1 M MgSO_4_, 0.1 M CaCl_2_, 10 mg/L vitamin B1 and 20 g/L glucose was used for mutagenesis screening and fermentation confirmation. For yeast concentration optimization, glucose concentration in M9 medium was changed to 40 g/L and various concentration of yeast extract (0, 1, 2, 3, 4 or 5 g/L) was added in the medium. *E. coli* XL1-Blue was used for plasmid construction, while *E. coli* BW25113 (F′) was used for feeding experiments and as parent strain for ARTP mutagenesis. The details of strains and plasmids were described in Table 2.

**Table 2:** Plasmids and strains used in this study

	Description	Source
Plasmids		
pUC19	*P*_*lac*_-*lacZα; colE1; amp*^*r*^	TransGen Biotech
pYK	*P*_*L*_*lacO*_*1*_; *LacI; colA; kan*^*r*^	[16]
pSA74	*P*_*L*_*lacO*_*1*_; pSC101*; cm^*r*^	[36]
pSA65	*P*_*L*_*lacO*_*1*_-*kivd*-*adhA; colE1; amp*^*r*^	[8]
pSA69	*P*_*L*_*lacO*_*1*_-*alsS*-*ilvC*-*ilvD; p15A; kan*^*r*^	[8]
pYH1	*P*_*bmoR*_-*bmoR; P*_*bmo*_-*gfp; colE1; amp*^*r*^	This study
pYH2	*P*_*bmoR*_-*bmoR; P*_*bmo*_-*lacZα; colE1; amp*^*r*^	This study
pYH3	*P*_*bmoR*_-*bmoR; P*_*bmo*_-lacZα; pSC101*; cm^*r*^	This study
pYH4	*P*_*bmoR*_-*bmoR; P*_*bmo*_-*lacZα; P*_*L*_*lacO*_*1*_-*kivd*-*adhA; colE1; amp*^*r*^	This study
pYH5	*P*_*bmoR*_-*bmoR; P*_*bmo*_-*lacZα; colA; cm*^*r*^	This study
pYH9	*P*_*bmoR*_-*bmoR; P*_*bmo*_-*gfp; P*_*L*_*lacO*_*1*_-*kivd*-*adhA; colE1; amp*^*r*^	This study
pYH10	*P*_*bmoR*_-*bmoR; P*_*bmo*_-*gfp; colA; cm*^*r*^	This study
pYH11	*P*_*bmoR*_-*bmoR; P*_*bmo*_-gfp; pSC101*; cm^*r*^	This study
Strains		
XL1-Blue	*recA1 endA1 gyrA96 thi*-*1 hsdR17 supE44 relA1 lac [F′ proAB lacI*^*q*^*ZΔM15 Tn10 (Tet*^*r*^*)]*	[8]
BW25113 (F′)	*rrnBT14ΔlacZWJ16hsdR514ΔaraBADAH33* *ΔrhaBADLD78F′ [traD36 proABlacIqZΔM15 Tn10(Tet* ^*r*^ *)]*	[8]
YH1	BW25113 (F′) with pYH1	This study
YH2	BW25113 (F′) with pYH2	This study
YHS1	BW25113 (F′) with pSA69 and pYH4	This study
YHS2	BW25113 (F′) with pSA69, pSA65 and pYH5	This study
YHS3	BW25113 (F′) with pSA69, pSA65 and pYH3	This study
YHS4	BW25113 (F′) with pSA65 and pYH5	This study
YHS5	BW25113 (F′) with pSA65 and pYH10	This study
YHS6	Mutant 10 with pSA69	This study

### DNA manipulation

Plasmids pUC19 from TransGen Biotech, pYK [16] and pSA74 [36] were used as templates to amplify genes *lacZα*, *colA* origin and pSC101* origin, respectively. Plasmids pSA65 and pSA69 were from previous study [8]. Plasmid pSA65 was used as template to amplify *colE1* origin. Gene *gfp* (accession number: AAX07425.1) was synthesized by OE-PCR. The *P*_*bmoR*_-*bmoR* and *P*_*bmo*_ were referenced from NCBI (accession number: AY093933.3). *P*_*bmoR*_-*bmoR* was synthesized by OE-PCR after codon optimization for *E. coli* and then ligated with *colE1* origin, *amp*^*r*^ and *P*_*bmo*_-*gfp* via Gibson Assembly, generating plasmid pYH1. To construct plasmid pYH2, *gfp* of pYH1 was replaced with *lacZα*. Replacing *colE1* origin and *amp*^*r*^ of pYH2 with pSC101* origin and *cm*^*r*^, respectively, generated plasmid pYH3. To create plasmid pYH5, pSC101* origin of pYH3 was substituted with *colA* origin. *P*_*bmoR*_-*bmoR*, *P*_*bmo*_-*lacZα*, *P*_*L*_*lacO*_*1*_-*kivd*-*adhA* which was amplified from pSA65, *colE1* origin and *amp*^*r*^ were assembled via Gibson Assembly to generate plasmid pYH4. Gene *lacZα* of pYH3, pYH4 and pYH5 were replaced with *gfp*, resulting in plasmids pYH11, pYH9 and pYH10, respectively. All the plasmids were sequenced by GENEWIZ company.

### Extracellular confirmation of BmoR-*P*_*bmo*_ biosensor based on GFP fluorescence

*Escherichia coli* BW25113 (F′) harboring plasmid pYH1 was pre-inoculated into 5 mL LB with 100 μg/mL ampicillin and then cultured at 37 °C overnight. Then, 50 μL of the seed culture was transferred into 950 μL fresh LB medium supplemented with different concentrations (0, 0.01, 0.1, 1, 10, 20, 40, 50 or 100 mM) of *n*-butanol, isobutanol and 3-MB, respectively, in 96-deep-well plates and the cultures were then left at 30 °C for 16 h. After that, GFP fluorescence and OD_600_ values were detected by microplate reader (BioTek Cytation 3). GFP fluorescence was measured using an excitation wavelength of 470 nm and an emission wavelength of 510 nm. GFP fluorescence value was normalized as GFP/OD_600_, and the background fluorescence of medium was subtracted. *K*_m_ values were estimated with OriginPro8.5 through non-linear regression of the Hill1 equation.

### Extracellular confirmation of BmoR-*P*_*bmo*_ biosensor based on blue–white screening

*Escherichia coli* BW25113 (F′) harboring the biosensor plasmid pYH2 were cultured overnight in 5 mL LB medium with 100 μg/mL ampicillin, and then 50 μL of seed culture was transferred into 5 mL fresh LB medium with different concentrations of isobutanol (0, 0.01, 0.1, 1, 10, 20, 40, 50 or 100 mM) feeding when OD_600_ reached 0.2–0.4. Cultures were then left at 30 °C for 24 h, and then 10 μL of the seed culture was diluted 5000 times and spread on the LB plate with 100 μg/mL X-gal, different concentrations of isobutanol and associated antibiotics. The plates were placed in a 30 °C incubator for 72 h.

### Verifying BmoR-*P*_*bmo*_ biosensor response to isobutanol produced by cell based on blue–white screening

Plasmids with different copies (pYH4, pYH5 and pYH3, high-copy, medium-copy and low-copy-number, respectively) were introduced into isobutanol-producing strain *E. coli* BW25113 (F′) (pSA65/pSA69). The single colonies were inoculated into 5 mL LB medium with appropriate antibiotics and grew at 37 °C until OD_600_ reached around 1.0. Then, 10 μL of the seed culture was diluted 5000 times and spread on the M9 plate with 100 μg/mL X-gal, 0.1 mM IPTG and associated antibiotics. The plates were placed in a 30 °C incubator for 72 h.

### ARTP mutagenesis and screening

The ARTP mutation system which could cause greater gene damage than traditional mutagenesis was employed to generate the mutation library [37]. *E. coli* BW25113 (F′), as initial strain, was pre-incubated into 5 mL LB medium and cultured at 37 °C for 4 h to reach its logarithmic phase. Then, 10 μL of the culture was transferred to stainless steel minidisc and subsequently exposed to ARTP with 60-s irradiation. The mutated cells were recovered in 3 mL fresh LB medium at 37 °C for 6 h and then stored in 15% glycerol for screening. For screening, the cells in mutagenesis library were transformed with plasmids pSA65 and pYH10. The single colonies were pre-incubated into 5 mL LB medium with 100 μg/mL ampicillin and 25 μg/mL chloromycetin at 37 °C for 12 h. 50 μL of the seed culture was added into 1 mL M9 medium with 0.1 mM IPTG in 96-deep-well plates. All 96-deep-well plates with cultures were incubated at 30 °C for 20 h. The fluorescence and OD_600_ values were measured by the microplate reader.

### Fermentation verification of the isobutanol overproduction strains in shake flask

The single colonies were pre-inoculated into 5 mL LB medium with associated antibiotics at 37 °C for 12 h. Then, 200 μL culture was inoculated into 20 mL M9 with 0.1 mM IPTG and various concentration of yeast extract in 250 mL screw cap conical flask and then left at 30 °C. Samples were taken every 12 h. Then, 200 μL of sample was added into 96-well plates for fluorescence and OD_600_ measurement. Production of alcohols was quantified by Agilent 6890 GC chromatograph equipped with flame ionization detector (Agilent Technologies, CA, USA). The separation of alcohols was carried out by a DB-FFAP capillary column (30 m × 0.32 mm × 0.25 μm; Agilent Technologies). For analysis of isobutanol, the GC oven temperature was initially held at 80 °C for 3 min, increased with a gradient of 115 °C/min until 230 °C, and kept at 230 °C for an additional 1 min. Nitrogen was used as the carrier gas. The injector and detector were maintained at 250 and 280 °C, respectively. Supernatant (1 μL) was sampled and injected at a split ratio of 1:30 and n-pentanol was used as internal standard.

### Culture medium for fed-batch fermentation

To evaluate the isobutanol-producing potential of the mutated strain which was screened out from the mutation library, the fermentation was performed in a 3-L bioreactor with 2 L working volume. The culture medium containing 40 g/L glucose, 3 g/L (NH_4_)_2_SO_4_, 14.6 g/L K_2_HPO_4_, 4 g/L KH_2_PO_4_, 2.2 g/L sodium citrate, 8 g/L yeast extract, 1.25 g/L MgSO_4_·7H_2_O, 0.1 g/L ampicillin, 0.05 g/L kanamycin, 0.025 g/L chloromycetin and 1 mL/L trace metal solution was used for bioreactor fermentation. Trace metal solution contained 14.1 g EDTA, 2.5 g CoCl_2_·6H_2_O, 15 g MnCl_2_·4H_2_O, 1.5 g CuCl_2_·2H_2_O, 3 g H_3_BO_3_, 2.1 g Na_2_MoO_4_·2H_2_O, 33.8 g Zn(CH_3_COO)_2_·2H_2_O and 80 g FeCl_3_·6H_2_O per liter. During the cultivation period, 1.5 L stock solution containing 500 g/L glucose, 1.25 g/L MgSO_4_·7H_2_O, 0.1 g/L ampicillin, 0.05 g/L kanamycin, 0.025 g/L chloromycetin and 0.1 mM IPTG was fed to the batch culture.

### Bioreactor culture conditions

The bioreactor was inoculated with 5% of overnight pre-culture and the cells grown at 37 °C with 1 vvm of air flow rate and 600 rpm of stirrer speed for 2 h. Then, 0.1 mM IPTG was added into the bioreactor and the temperature was changed to 30 °C to induce the expression of the enzymes in isobutanol production pathway. The pH was controlled at 6.8 by automatic addition of ammonia solution (25%). After induction of 12 h, the air flow rate was increased from 1 to 3 vvm in order to strip out isobutanol from the broth. The evaporated isobutanol was condensed by a condenser and subsequently the generated liquid isobutanol flowed into collection bottle A which was cooled with ice (Fig. 7). The residual uncondensed isobutanol was collected into bottle B and C which containing 800 mL water and were also cooled with ice (Fig. 7). The samples were taken to determinate the biomass, glucose concentration and isobutanol titer.
